# Understanding real and mythical cancer risk factors: Insights from a university-based study

**DOI:** 10.1371/journal.pone.0336102

**Published:** 2025-11-26

**Authors:** Senay Yildirim-Kahriman, Gulay Yildirim, Selmin Kose

**Affiliations:** 1 Faculty of Health Sciences, Demiroglu Bilim University, Istanbul, Turkey; 2 Department of Nursing, Kesan Hakki Yoruk School of Health, Trakya University, Edirne, Turkey; 3 Department of Nursing, Faculty of Health Sciences, Biruni University, Istanbul, Turkey; Freelance Medical Research and Writing, UNITED KINGDOM OF GREAT BRITAIN AND NORTHERN IRELAND

## Abstract

Despite extensive research on cancer etiology, many cancers remain associated with preventable risk factors. Raising public awareness of these factors is critical for promoting preventive behaviors. This study aimed to assess university students’ awareness of both scientifically validated and mythical cancer causes, to identify sociodemographic and health/lifestyle-related factors associated with awareness levels, and to examine the relationship between awareness of evidence-based and mythical cancer risk factors. A cross-sectional survey was conducted among 896 students from three Universities in Turkey. Data were collected using a Personal Information Form, the Cancer Awareness Measure (CAM) and Cancer Awareness Measure – Mythical Causes Scale (CAM-MYCS), which evaluates belief in widely held but scientifically unsubstantiated cancer causes (e.g., microwave ovens, stress, artificial sweeteners, electromagnetic frequencies). Statistical analyses included descriptive statistics, Spearman’s correlation, and multiple linear regression. Participants demonstrated significantly higher awareness of scientifically established cancer causes (70.7%) compared to mythical ones (10.2%). A moderate negative correlation was observed between CAM and CAM-MYCS scores (r = −0.511; p < 0.001), indicating that increased awareness of real causes is associated with decreased belief in myths. Regression analysis revealed that CAM scores were significantly predicted by academic year, being in the normal Body Mass Index (BMI) spectrum, and by having attended an oncology course (R² = 0.142; p < 0.001), whereas CAM-MYCS scores were predicted by academic year and high BMI values (R² = 0.072; p < 0.001). These findings highlight the need for targeted educational interventions aimed at both enhancing awareness of factual cancer risk factors and correcting persistent misconceptions. The results provide valuable insights for university-based public health initiatives seeking to improve cancer literacy and promote preventive health and lifestyle behaviors among young adults.

## Introduction

Cancer encompasses a group of diseases characterized by uncontrolled cellular proliferation with the potential for invasion and metastasis to distant sites. Broadly, cancers are categorized into two main types: solid tumors—such as those of the breast, lung, colon, and prostate—and hematologic malignancies, including leukemias, lymphomas, and myelomas. Epidemiological data reveal marked differences in the incidence and prevalence of various cancers across genders and age groups, notably between pediatric and adult populations [1]. These disparities in cancer incidence across demographic groups can be attributed, in part, to varying distributions of intrinsic and environmental risk factors [2]. Cancer risk factors can be stratified into two primary domains: patient-related (intrinsic) and environmental (extrinsic) factors. Patient-related factors include age, sex, genetic susceptibility, and hormonal status, whereas environmental risk factors encompass modifiable exposures such as tobacco use, infections (e.g., human papillomavirus [HPV], hepatitis viruses), ultraviolet (UV) radiation, occupational hazards, dietary patterns, and physical inactivity. Among these, environmental factors are particularly amenable to prevention strategies [3–5].

The Cancer Awareness Measure (CAM), developed by Stubbings et al. in 2009, serves as a validated instrument to assess public awareness of evidence-based cancer risk factors. The selected items in CAM reflect both scientific validity and public health relevance [6]. In contrast, the Cancer Awareness Measure–Mythical Causes Scale (CAM-MYCS), introduced by Smith et al. in 2018, evaluates belief in widely held but scientifically unsubstantiated causes of cancer —such as the use of microwave ovens, genetically modified foods, and exposure to electromagnetic fields [7]. Both instruments have been extensively used in international research to assess public understanding and misconceptions regarding cancer etiology [8–12].

While awareness of scientifically supported cancer risk factors is fundamental for fostering preventive behaviors, the persistence of misinformation and belief in mythical causes can undermine public health efforts [9,13]. In this context, the present study aims to comprehensively assess awareness levels of both validated and mythical cancer risk factors among paramedical university students—a population likely to play a critical role in future health education initiatives. Specifically, the study seeks to (i) evaluate the extent of awareness of evidence-based and mythical cancer risk factors, (ii) identify sociodemographic and health/lifestyle related factors associated with awareness levels, and (iii) examine the correlation between awareness of validated and mythical factors. By addressing these objectives, the study intends to highlight key knowledge gaps and provide evidence to guide targeted educational interventions that may enhance cancer literacy in the broader population.

## Materials and methods

### Study design, setting and population

This study employed a cross-sectional descriptive and analytical design. It was conducted between January and March 2024. The reporting of this study followed the STROBE (Strengthening the Reporting of Observational Studies in Epidemiology) guidelines, and the corresponding STROBE checklist is provided as Supporting Information (S1 Checklist).

The research was conducted at Faculties of Health Sciences at three universities in Turkey—one public and two private. Within these faculties, the departments of Nursing, Midwifery, Nutrition and Dietetics, Physiotherapy and Rehabilitation, Child Development, Speech and Language Therapy, Occupational Therapy, and Audiology were included in the study scope. The participants were undergraduate students enrolled in first through fourth academic years across these departments.

Approximately 4,300 undergraduate students were enrolled at the participating faculties at the time of the study. Among them, 78% were female and 22% were male. The study population consisted of students who were accessible during the data collection period and who voluntarily agreed to participate in the research.

Eligibility criteria included being 18 years of age or older, being enrolled in a Faculty of Health Sciences, and providing voluntary consent to participate. Students under 18 years of age or those with visual impairments that prevented survey participation were excluded.

Although the total number of students enrolled in the participating faculties was approximately 4,300, this number was an estimate and not a precisely defined population size. Therefore, the sample size was calculated using the standard formula for an unknown (infinite) population: n=[Z^2^xp(1-p)]/e^2^ where Z = 1.96 (for a 95% confidence level), p = 0.5 (maximum variability), and e = 0.05 (margin of error). Based on this calculation, a minimum of 384 participants was required. The final sample of 896 participants exceeded this threshold, ensuring sufficient statistical power and representativeness for inferential analysis.

### Sampling procedure

A non-probability, convenience sampling method was used to recruit participants. This method was chosen due to its practicality and feasibility in accessing a large number of university students during a limited data collection period, especially in an online survey.

### Study variables and measurement tools

To ensure data quality, standardized procedures were followed throughout the data collection process. Informed consent was obtained digitally from all participants. Data were collected using previously validated instruments. Although no pilot study was conducted, all measurement tools were based on validated sources. Continuous monitoring of data entry was performed to minimize errors and maintain accuracy. Participant anonymity and confidentiality were strictly preserved. Data were collected online via a Google Form using a structured questionnaire composed of three sections (see Supporting Information S2 Survey Instrument). All participants who provided consent first completed the personal and demographic items, followed by the CAM and CAM-MYCS scales, which were grouped under the heading “Cancer Awareness.” The section on health and lifestyle behaviors was administered after these scales. Personal Information Form collected both socio-demographic information—including age, gender, marital status, and income level—and descriptive characteristics, such as academic department, year of study, prior coursework in oncology, and whether the participant had a family member diagnosed with cancer.

CAM, an 11-item, 5-point Likert-type scale assessing awareness of scientifically validated cancer risk factors such as tobacco use, obesity, alcohol consumption, physical inactivity, and excessive sun exposure. In line with the original scoring method, responses of “Strongly Agree” and “Agree” were coded as correct (1 point), while other responses were coded as incorrect (0 points). The total score ranged from 0 to 11, with higher scores indicating greater awareness. As no cut-off points have been established for the CAM scale in the literature, total scores were treated as continuous variables. In the current study, the scale demonstrated acceptable internal consistency with a Cronbach’s alpha of 0.732, comparable to the original study (α = 0.77) [6]. CAM-MYCS, a 12-item, 5-point Likert scale developed to assess belief in commonly held but scientifically unsupported cancer myths—such as the use of microwave ovens, stress, genetically modified foods, and mobile phones. According to the validated scoring system, “Strongly Disagree” and “Disagree” were scored as correct (1 point), and all other responses were scored as incorrect (0 points), yielding a total score between 0 and 12. Higher scores reflect lower belief in mythical causes and, thus, greater awareness. Similar to the CAM scale, the CAM-MYCS does not include predefined cut-off values, and scores were analyzed as continuous variables. In this study, the scale demonstrated acceptable reliability (Cronbach’s alpha = 0.731), consistent with the original validation by Smith et al. (2018) (α = 0.86) [7]. The Turkish version was previously adapted and validated by Coskun (2020) using back-translation and content validity indexing methods [14].

Health and Lifestyle Behaviors Form included questions on Body Mass Index (BMI) smoking (using of traditional cigarettes), physical activity levels, fruit and vegetable intake, and, alcohol consumption. This set of behavioral and lifestyle factors was collected to examine their potential associations with cancer awareness levels, as measured by the CAM and CAM-MYCS questionnaires. These variables are recognized in the literature [9] as modifiable risk factors for chronic diseases, including several types of cancer. BMI was calculated using self-reported current height and weight, based on the formula: weight in kilograms divided by height in meters squared. Participants were categorized as belonging to the normal weight (18.5–24.9), overweight (25.0–29.9), or obese (≥30) categories according to the World Health Organization (WHO) classification of BMI [15]. Smoking status was assessed by asking participants whether they currently smoked, or had never smoked, and classified accordingly. Physical activity was measured by asking how many days per week participants engaged in activities lasting at least 30 minutes per day that increased their breathing rate [9]. Physical activity levels were evaluated according to 2020 WHO recommendations, which state that a minimum of 150 minutes of moderate activity is advised per week [16]. The level of fruit and vegetable consumption was measured by asking participants how many servings (1 serving = 80g; one small apple approximately 90 g) they consumed daily [9]. Fruit and vegetable consumption of at least five servings (approximately 400g) per day was considered as meeting the recommended level [17]. Alcohol use was assessed and quantified by asking how many days per week and how many units per day participants consumed alcohol (1 unit was considered as 1 can/bottle of beer, 1 glass of wine, or 45 ml of spirits such as whiskey or vodka).

### Operational definitions

In this study, cancer awareness was operationalized through two validated instruments: the CAM (Cancer Awareness Measure), which assesses recognition of scientifically established cancer risk factors, and the CAM-MYCS (Mythical Causes Scale), which evaluates beliefs in unvalidated or mythical cancer causes. Factors considered to be associated with cancer awareness were selected among sociodemographic characteristics (age, gender, marital status, income, department, year of study), oncology course attendance, family history of cancer, and health/lifestyle behaviors (BMI, smoking, physical activity, diet, and alcohol use).

### Statistical analysis

Data were analyzed using SPSS version 22.0. The normality of distribution was tested using the Kolmogorov-Smirnov test. As not all variables used in inferential analyses followed a normal distribution, nonparametric tests were applied where appropriate. Descriptive data such as frequencies, percentages, and mean scores are presented in Tables 1 and 2. Mann–Whitney U tests were used for comparisons between two groups, and Kruskal–Wallis tests were used for comparisons among three or more groups. The relationship between CAM and CAM-MYCS scores was examined using Spearman’s correlation test [18].

**Table 1:** Sociodemographic characteristics of the sample.

Characteristics^∞^		N	%	95% CI
**Age** (Mean:20.63 ± 2.6)	18 years old	106	11.8	9.9-14.1
	19-24 years old	756	84.4	81.9-86.6
	25-45 years old	34	3.8	2.7-5.3
**Gender**	Women	763	85.2	82.7-87.3
	Men	133	14.8	12.7-17.3
**Marital status**	Married	11	1.2	0.7-2.2
	Single	885	98.8	97.8-99.3
**Income**ª	Low	130	14.5	12.4-17.0
	Middle	737	82.3	79.6-84.6
	High	29	3.2	2.3-4.6
**Study field**	Nursing	403	45	41.7-48.2
	Midwifery	167	18.6	16.2-21.3
	Nutrition and Dietetics	47	5.2	4.0-6.9
	Physiotherapy and Rehabilitation	97	10.8	9.0-13.0
	Child Development	31	3.5	2.4-4.9
	Speech and Language Therapy	96	10.7	8.9-12.9
	Occupational therapy	39	4.4	3.2-5.9
	Audiology	16	1.8	1.1-2.9
**Academic year in university education**	I	367	41.0	37.8-44.2
	II	259	28.9	26.0-32.0
	III	142	15.8	13.6-18.4
	IV	128	14.3	12.1-16.7
**Have you taken a course on oncology?** ^•^	Yes	127	14.2	12.0-16.6
	No	769	85.8	83.4-88.0
**Do you have a first or second degree relative diagnosed with cancer?** ^¥^	Yes	380	42.4	39.2-45.7
	No	516	57.6	54.3-60.8

∞ Data on race and ethnicity were not collected, as such classifications are not routinely used in health-related academic research in Turkey.

ªLow (0–25.000 Turkish Lira (₺)); Middle (25.001 ₺-60.000 ₺); High (60.001 ₺ and above).

• Elective course.

¥ First-degree relatives include parents, siblings, and children; second-degree relatives include grandparents, aunts/uncles, and nieces/nephews.

**Table 2:** Means scores of CAM and CAM-MYCS.

Scales	Mean	± SD	Min	Max
**CAM Questions**				
1. Active smoking is a risk factor for cancer.	0.97	±0.2	0	1
2. Drinking alcohol is a risk factor for cancer.	0.88	±0.3	0	1
3. Passive smoking is a risk factor for cancer.	0.81	±0.4	0	1
4. Sunburn is a risk factor for cancer	0.74	±0.4	0	1
5. Human Papilloma Virus is a risk factor for cancer.	0.72	±0.4	0	1
6. Red processed meat products are a risk factor for cancer.	0.72	±0.4	0	1
7. Obesity is a risk factor for cancer.	0.66	±0.5	0	1
8. Having a relative diagnosed with cancer is a risk factor for cancer..	0.78	±0.4	0	1
9. Inadequate physical activity (exercise) is a risk factor for cancer.	0.55	±0.5	0	1
10. Aging is a risk factor for cancer.	0.40	±0.5	0	1
11. Inadequate fruit and vegetable consumption is a risk factor for cancer.	0.50	±0.5	0	1
CAM Total (0–11 points range)	7.74	±2.4	0	11
CAM Total (%)	70.72	±21.83	9.10	100
**CAM-MYCS Questions**				
1. Exposure to electromagnetic frequencies (low frequency non-ionizing radiation such as wifi, tv, radio)	0.036	±0.9	0	1
2. Eating foods that contain additives	0.26	±0.1	0	1
3. Living near power lines	0.091	±0.3	0	1
4. Feeling stressed	0.028	±0.2	0	1
5. Eating foods containing artificial sweeteners	0.022	±0.1	0	1
6. Using cleaning products	0.114	±0.3	0	1
7. Using a mobile phone	0.173	±0.4	0	1
8. Eating genetically modified foods	0.013	±0.1	0	1
9. Using containers containing aerosols	0.084	±0.3	0	1
10. Physical trauma such as hitting or crushing	0.381	±0.5	0	1
11. Using a microwave oven	0.147	±0.3	0	1
12. Drinking from a plastic bottle	0.115	±0.3	0	1
CAM-MYCS Total (0–12 points range)	1.299	±1.7	0	11
CAM-MYCS Total	10.2473	±14.42	0.00	91.66

To identify predictors of cancer awareness, two separate multiple linear regression analyses were conducted. The dependent variables were the total scores obtained from the CAM (range: 0–11) and CAM-MYCS (range: 0–12) scales. These total scores represent the number of correct responses given by each participant across all items in each scale. Independent variables included: age, gender, marital status, income level, academic department, year of study, completion of an oncology-related course, family history of cancer (first- or second-degree relatives), and behavioral/lifestyle factors (BMI category, smoking status, physical activity level, fruit and vegetable intake, and alcohol use). Before conducting the analyses, regression assumptions were assessed through residual plots and multicollinearity diagnostics (Variance Inflation Factor, VIF). Model fit was evaluated using R², adjusted R², F-tests, and standardized beta coefficients (β). A p-value <0.05 was considered statistically significant.

### Patient and public involvement

Patients or members of the public were not involved in the design, conduct, reporting, or dissemination plans of this research. The study was designed by the research team based on existing literature and public health priorities.

### Ethical considerations

The study received ethical approval from the Non-Interventional Clinical Research Ethics Committee of Biruni University (Decision No. 2023/85–57, dated 22.12.2023). All procedures were conducted in accordance with the Declaration of Helsinki. Informed consent was obtained from all participants prior to data collection via a digital form.

## Results

### Sociodemographic factors

Of the total participants, 85.2% were female and 14.8% male. The majority were between the ages of 18 and 45 years. Most participants were single and enrolled in the departments of Nursing, Midwifery, and Physiotherapy and Rehabilitation. More than half of the sample reported a moderate income level. First- and second-year students constituted the largest share of the study population. The sociodemographic characteristics of the participants included in the study are presented in Table 1.

### Awareness for accepted and mythical cancer risk factors

**Fig 1:**
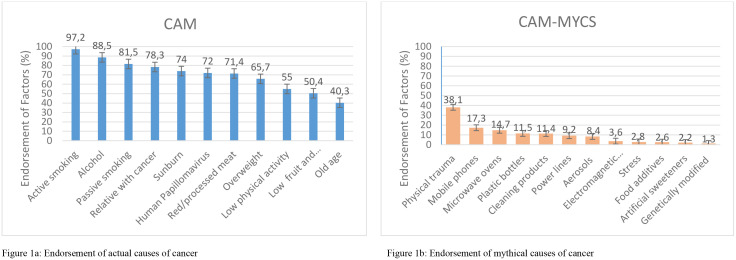
Participants’ endorsement of actual and mythical cancer risk factors. (a) Endorsement of actual causes of cancer. (b) Endorsement of mythical causes of cancer.

More than four-fifths of the participants endorsed the established cancer risk factors listed in the CAM, including active smoking (97.2%), alcohol consumption (88.5%), and passive smoking (81.5%). At least half of the participants also identified insufficient fruit and vegetable intake (50.4%) and lack of physical activity (55%) as cancer risk factors. The least endorsed risk factor was aging, with only 40.3% recognizing it as a cancer risk factor (Fig 1a). The CAM-MYCS item most accurately identified by participants as not being a proven cancer risk factor was physical trauma, such as being hit or crushed (38.1%). Fewer than 10% of participants correctly rejected the following CAM-MYCS items as cancer risk factors: genetically modified foods (1.3%), artificial sweeteners (2.2%), foods containing additives (2.6%), stress (2.8%), electromagnetic frequencies (3.6%), aerosol sprays (8.4%), and power lines (9.2%). Overall, the percentage of correct responses for all other CAM-MYCS items, except for the physical trauma item, remained below 18% (Fig 1b).The mean score of responses to the CAM items was 70.72 ± 21.8, while the mean score of responses to the CAM-MYCS items was 10.25 ± 14.42 (Table 2).

Participants aged 25 years and older had significantly higher CAM median scores than younger age groups (p = .000; η² = .05). Married participants scored significantly higher on the CAM median scores compared to unmarried participants (p = .002; r = .10). Students enrolled in the midwifery department had higher CAM median scores than those in departments other than nursing and audiology (p = .000; η² = .053). CAM median scores were significantly higher among second- and fourth- year students compared to first- and third- year students (p = .000; η² = .10). No significant differences were found in CAM median scores based on participants’ gender or income level (p = .209 and p = .620, respectively). Those who had taken an oncology-related course had higher CAM median scores than those who had not (p = .000; r = .02). Participants with a first- or second-degree relative diagnosed with cancer also had higher CAM scores (p = .042; r = .06) (Table 3). Regarding behavioral and lifestyle factors, participants with a normal Body Mass Index (BMI) scored higher on the CAM median scores than those who were overweight or obese (p = .000; r = .17). In addition, those who consumed the recommended amount of fruits and vegetables (≥400g/day) had higher CAM median scores (p = .004; r = .10). However, no significant differences in CAM median scores were found based on smoking status, physical activity level, or alcohol consumption (p = .322, p = .286, and p = .872, respectively) (Table 4). Participants who were 18 years old had significantly higher CAM-MYCS median scores than those in older age groups (p = .000; η² = .03). Unmarried students had higher CAM-MYCS median scores than their married peers (p = .013; r = .08). Students from the midwifery department had lower CAM-MYCS median scores than students in departments other than audiology (p = .000; η² = .02). First-year students scored higher on the CAM-MYCS than those in the second, third, and fourth years (p = .000; η² = .02). Those who had not taken an oncology course scored significantly higher than those who had (p = .000; r = .14). No significant differences in CAM-MYCS median scores were found based on participants’ gender, income level, or having a first- or second-degree relative diagnosed with cancer (p = .455, p = .208, and p = .383, respectively) (Table 3). With respect to behavioral and lifestyle factors, overweight or obese participants had higher CAM-MYCS median scores than those with normal BMI (p = .002; r = .10), and participants who did not meet fruit and vegetable intake recommendations scored higher than those who did (p = .012; r = .08). No significant differences were found in CAM-MYCS median scores based on smoking status, physical activity, or alcohol consumption (p = .611, p = .254, and p = .656, respectively) (Table 4).

**Table 3:** CAM and CAM-MYCS scores stratified by sociodemographic characteristics, oncology course attendance, and family history of cancer.

Characteristic	CAM Scores		CAM-MYCS Scores	
	Median (IQR)	Z/*X*^2^ p	Median (IQR)	Z/ *X* ^2^ p
**Age**				
18 years old	54.50 (45.60-65.87)	**47.06** ^*#*^ **<0.001**	8.33 (0.0-8.33)	**26.73** ^***#***^ **<0.001**
19-24 years old	72.70 (54.50-90.90)		0.00 (0.0-0.0)	
25-45 years old	90.90 (63.60-100.0)		0.00 (0.0-0.0)	
**Gender**				
Women	72.70 (54.50-90.90)	-1,256^*&*^ 0.209	8.33 (0.0-8.33)	-0.747^*&*^ 0.455
Men	72.70 (54.50-90.90)		0.00 (0.0-0.0)	
**Marital status**				
Married	90.90 (90.90-100.0)	**-3.090** ^*&*^ **<0.01**	0.00 (0.0-0.0)	**-2.495** ^***&***^ **<0.05**
Single	72.70 (54.50-72.70)		8.33 (0.0-8.33)	
**Income**				
Low	72.70 (54.50-90.90)	0.957^*#*^ 0.620	8.33 (0.0-8.33)	3.143^*#*^ 0.208
Middle	72.70 (72.70-90.90)		8.33 (0.0-8.33)	
High	72.70 (72.70-90.90)		8.33 (0.0-8.3)	
**Study field**				
Nursing	72.70 (54.50-81.80)	**54.40** ^*#*^ **<0.001**	8.33 (0.0-16.66)	**26.34** ^***#***^ **<0.001**
Midwifery	81.80 (72.70-100.0)		0.00 (0.0-8.33)	
Nutrition and Dietetics	68.15 (54.50--81.80)		8.33 (0.0-8.18.74)	
Physiotherapy and Rehabilitation	63.60 (45.60-81.80)		8.33 (0.0-16.66)	
Child Development	63.60 (54.50-63.60)		8.33 (0.0-16.66)	
Speech and Language Therapy	63.60 (45.60-90.90)		8.33 (0.0-25.00)	
Occupational therapy	63.60 (45.60-72.70)		8.33 (0.0-25.00)	
Audiology	77.25 (457.82-90.90)		4.16 (0.0-14.58)	
**Academic year in university education**				
I	63.60 (45.60-81.80)	**93.67** ^*#*^ **<0.001**	8.33 (0.0-25.0)	**52.53** ^***#***^ **<0.001**
II	81.80 (63.60-100.0)		0.00 (0.0-8.33)	
III	72.70 (54.50-90.90)		4.16 (0.0-4.16)	
IV	81.80 (63.60-90.90)		0.00 (0.0-0.0)	
**Have you taken a course on oncology?**				
Yes	86.35 (72.70-100.0)	**-6.381** ^*&*^ **<0.001**	0.00 (0.0-0.0)	**-4.180** ^***&***^ **<0.001**
No	72.70 (54.50-90.90)		8.33 (0.0-8.33)	
**Do you have a first or second degree relative diagnosed with cancer?**				
Yes	72.70 (54.50-72.70)	**-2,029** ^*&*^ **<0.05**	8.33 (0.0-8.33)	-0.872^*&*^ 0.383
No	72.70 (54.50-72.70)		8.33 (0.0-8.33)	

, ^#^Kruskal-Wallis test (**X** ^**2**^), ^&^Mann-Whitney test.

Only statistically significant differences are marked with an asterisk. (*p < 0.05; **p < 0.01, ***p < 0.001). Non-significant comparisons are included for completeness.

**Table 4:** CAM and CAM-MYCS scores stratified by lifestyle and health behavior factors.

Characteristic	n	%	CAM Scores		CAM-MYCS Scores	
			Median (IQR)	Z p	Median (IQR)	Z p
**BMI**						
<25 kg/m^2^	693	77.3	72.70 (54.50-90.90)	−**5.062**^*&*^ **<0.001**	0.00 (0.0-16.66)	**−3.135** ^*&*^ **<0.01**
≥25 kg/m^2^	203	22.7	63.60 (45.60-90.90)		8.33 (0.0-16.66)	
**Smoking**						
Yes	202	22.5	72.70 (54.50-90.90)	−0.989^*&*^ 0.322	8.33 (0.0-16.66)	−0.509^*&*^ 0.611
No	694	77.5	72.70 (54.50-90.90)		8.33 (0.0-16.66)	
**Physical activity**						
≥150 min of exercise per week	49	5.5	72.70 (54.50-81.80)	−1.066^*&*^ 0.286	8.33 (0.0-16.66)	−1.141^*&*^ 0.254
<150 min of exercise per week	847	94.5	72.70 (54.50-90.90)		8.33 (0.0-16.66)	
**Fruit/vegetable consumption**						
≥5 portions per day	48	5.4	81.80 (65.87-90.90)	**−2,853** ^*&*^ **<0.01**	0.00 (0.0-8.33)	**−2.504** ^*&*^ **<0.05**
<5 portions per day	848	94.6	72.70 (54.50-90.90)		8.33 (0.0-16.66)	
**Alcohol consumption**						
≥14 units per week	10	1.1	63.60 (54.50-90.90)	−0.162^*&*^ 0.872	4.16 (0.0-16.66)	−0.445^*&*^ 0.656
<14 units per week	886	98.9	72.70 (54.50-90.9)		8.33 (0.0-16.66)	

& Mann-Whitney test.

Only statistically significant differences are marked with an asterisk. (*p < 0.05; **p < 0.01, ***p < 0.001). Non-significant comparisons are included for completeness.

### Correlation between CAM and CAM-MYCS scores

A significant moderate negative correlation was found between the CAM and CAM-MYCS scales (r = –.511; p = .000). As participants’ awareness of actual cancer risk factors increased, their awareness of myths related to cancer risk factors decreased.

### Result of regression analysis of CAM and CAM-MYCS associated factors

The multiple linear regression model predicting CAM scores was significant (F = 14.57, p < .001; R² = .142, Adjusted R² = .132). Significant predictors included: grade level (β = .239, p < .001), oncology course attendance (β = .106, p = .002), and normal BMI (β = −.156, p < .001). The CAM-MYCS model was also significant (F = 6.85, p < .001; R² = .072, Adjusted R² = .062). Significant predictors were: grade level (β = −.179, p < .001) and high BMI (β = .087, p = .008). Cohen’s f² was 0.165 for the CAM model and 0.078 for the CAM-MYCS model. (Table 5).

**Table 5:** Regression results for factors predicting CAM and CAM-MYCS scores.

Variables	CAM (Total ccore, range: 0–11)			CAM-MYCS (Total score, range 0–12)			VIF
	B	β	p Değeri	B	β	p Değeri	
**Age**	0.044	0.047	0.172	−0.026	−0.040	0.265	1.236
**Gender**	0.140	0.020	0.531	0.222	0.045	0.174	1.064
**Academic year in university education**	1.193	0.239	0.000	−0.628	−0.179	0.000	1.328
**Oncology education** **Status**	0.743	0.106	0.002	−0.273	−0.055	0.111	1.141
**Family history of cancer in first and/or second degree relatives**	0.248	0.050	0.113	−0.154	−0.044	0.180	1.022
**BMI**	−0.912	−0.156	0.000	0.358	0.087	0.008	1.020
**Smoking (using of traditional cigarettes)**	−0.094	−0.016	0.623	0.121	0.029	0.387	1.079
**Physical activity**	−0.307	−0.028	0.372	0.491	0.064	0.051	1.026
**Fruit/vegetable consumption**	0.593	0.055	0.086	−0.309	−0.040	0.221	1.034
**Alcohol consumption**	−0.635	−0.027	0.391	−0.387	−0.024	0.476	1.038

“Note: B = Unstandardized regression coefficient, Beta = Standardized coefficient, p = Significance level, VIF = Variance Inflation Factor.”

As the independent variables are identical in both models, the VIF values remain the same for CAM and CAM-MYCS.

## Discussion

The main findings of this study are closely aligned with its primary aims. First, the majority of participants (70.72%) demonstrated a good level of awareness regarding scientifically validated cancer risk factors, whereas only 10.25% showed similar awareness toward mythical cancer causes, indicating a substantial knowledge gap. Second, participants with higher awareness of validated risk factors were significantly less likely to endorse mythical causes, suggesting a possible inverse relationship between scientific literacy and susceptibility to misinformation. Finally, in the multivariate analysis, variables such as academic year, prior oncology education, and BMI category emerged as significant predictors of awareness levels, reinforcing the importance of formal education and personal health context in shaping cancer-related knowledge.

### Awareness for evidence-based and mythical cancer risk factors

Regarding evidence-based risk factors, over half of the participants accurately identified known contributors to cancer, with the exception of aging. Among these, smoking—acknowledged as the leading cause of lung cancer and linked to 12–17 other cancer types [19]—was the most widely recognized. This finding is consistent with previous literature, both employing and not employing the CAM and CAM-MYCS scales, in which smoking has emerged as the most widely recognized cancer risk factor [9–11,20]. Similarly, a relatively high level of awareness was observed for HPV, identified by 72% of respondents as a cancer risk factor. Although HPV is primarily linked to cervical cancer, its association with head and neck malignancies is also well documented [21], suggesting participants had a commendable level of knowledge on this topic. In contrast, lifestyle-related factors such as obesity (65.7%) and physical inactivity (55%) were less frequently identified as cancer risks. This finding aligns with earlier literature showing a tendency to underestimate the impact of modifiable behaviors on cancer development [9,10,22,23]. These behaviors contribute to cancer through various biological pathways, including hormonal imbalance, metabolic dysfunction, insulin resistance, and chronic inflammation [24], and are classified among the preventable causes of cancer [25]. Notably, only 50% of participants recognized low fruit and vegetable consumption as a risk factor—making it one of the least acknowledged in our sample. This is in line with previous findings, where dietary factors often received the lowest recognition [9–11]. Aging was identified as the least known cancer risk factor, despite its fundamental biological role in carcinogenesis. Some researchers argue that while approximately one-third of cancer risk is attributable to modifiable environmental factors, the remaining majority results from intrinsic biological processes, such as cumulative stem cell divisions and random DNA replication errors [26]. From this perspective, aging represents a non-modifiable but inevitable risk factor—sometimes termed the “bad luck” component of cancer. However, more recent models posit that modifiable and non-modifiable factors are not mutually exclusive, and that decelerating the aging process through behavioral and medical interventions may reduce cancer risk by limiting DNA damage and modulating stem cell activity [27]. These findings underscore the importance of raising public awareness—particularly among younger populations—about aging as a cancer risk factor, and integrating this knowledge into prevention strategies.

Awareness of mythical cancer risk factors was notably low in our sample. Fewer than 18% of participants correctly identified all CAM-MYCS items—excluding the physical trauma item—as myths, a figure even lower than those reported in previous studies that also documented limited awareness of cancer-related misconceptions [9,10,20]. This suggests that university students in our study faced difficulty distinguishing evidence-based risk factors from commonly held but scientifically unfounded beliefs. One plausible explanation is the generalized perception that “everything causes cancer,” a cognitive bias that may lead participants to endorse items on both the CAM and CAM-MYCS scales without critical evaluation [10]. This bias is concerning, as it not only propagates misinformation but also blurs the distinction between real and mythical risks, ultimately complicating individuals’ ability to make informed health decisions. While low awareness of validated cancer risk factors is known to reduce engagement in preventive behaviors, belief in myths may be equally harmful. Misidentifying myths as real threats can divert attention from evidence-based preventive strategies—such as reducing alcohol intake or increasing physical activity—and may heighten cancer-related anxiety. Enhancing public understanding of scientifically supported risk factors is therefore essential. Educational interventions that improve individuals’ ability to discern between factual and mythical causes can foster more effective risk-reducing behaviors and mitigate misinformation. In our study, artificial sweeteners and genetically modified (GM) foods were the most commonly believed mythical cancer risk factors. This finding may be attributed to culturally specific narratives in Turkey, misinformation spread through social media platforms, and widespread skepticism toward industrial food production.

The overall mean CAM score in this study was moderately high, indicating a fair level of awareness regarding evidence-based cancer risk factors among participants. In contrast, the low mean CAM-MYCS score reflects poor ability to accurately reject mythical causes of cancer. This discrepancy suggests that while students may be familiar with established risk factors, they are considerably less adept at identifying misinformation or myths. Similar findings in previous studies have also highlighted this gap, emphasizing the need for integrated educational strategies that simultaneously promote evidence-based knowledge and critical thinking to combat cancer-related misconceptions [9,10,20].

### Sociodemographic and health/lifestyle factors associated with cancer awareness

When examined in relation to sociodemographic characteristics, the sample predominantly comprised a young population. Participants aged 25 and above demonstrated significantly greater accuracy in identifying validated cancer risk factors. Conversely, those under the age of 18 showed higher accuracy in recognizing mythical cancer risk factors (p < .001). This inverse trend suggests that younger participants may have greater exposure to digital health content, online awareness campaigns, and updated curricula, which may contribute to improved recognition of misinformation. Similar findings were reported by Shahab et al. (2018), who found that individuals under the age of 30 were more likely to correctly identify both evidence-based and mythical cancer causes [9]. In contrast, Munir et al. (2022) found no significant association between age and awareness levels [10]. Marital status was also associated with differing patterns of awareness. In our study, married participants demonstrated significantly higher scores on the CAM scale, while single participants performed better on the CAM-MYCS scale. These findings differ from Munir et al. (2022), who reported greater accuracy among single individuals in identifying actual cancer risk factors, and from Shahab et al. (2018), who found no significant association between marital status and awareness [9,10]. Participants who had taken a course in oncology demonstrated significantly higher levels of awareness regarding both validated and mythical cancer risk factors (p < .001, p < .001 respectively). The regression results support these findings. The likely contributors include curriculum content focused on evidence-based learning, which strengthens both theoretical understanding and practical health literacy. Given persistent gaps—particularly in distinguishing myths from factual risks—integrating cancer literacy into university curricula appears essential. Peer-led workshops, expert seminars, and digital campaigns may support myth-correction efforts, while broader public health interventions aimed at combating misinformation could further improve cancer awareness among young adults.

Participants with a normal BMI demonstrated significantly higher awareness of validated cancer risk factors compared to those classified as overweight or obese. This finding aligns with previous research suggesting that individuals maintaining a healthy weight are more likely to engage in preventive health behaviors and be receptive to health-related information [10,28]. One possible explanation is that health-conscious individuals may actively seek and internalize evidence-based information, which contributes both to healthier weight status and greater awareness of cancer risks.

Similarly, participants who met the recommended daily intake of fruits and vegetables exhibited greater awareness of validated cancer risk factors. This may reflect greater exposure to nutritional and preventive health education among those who adhere to dietary guidelines, as dietary behaviors often correlate with health literacy levels [9]. Therefore, a higher level of cancer awareness in this group might stem from broader engagement with health-promoting knowledge and practices.

### Correlation between measures of awareness for evidence-based and mythical risk factors

Participants demonstrated greater awareness of scientifically validated cancer risk factors compared to unproven myths, with a significant negative correlation observed between the two domains. This pattern is consistent with previous findings from populations in England [9], Pakistan [10], and Spain [11], suggesting that individuals who are more informed about evidence-based risks may be less adept at identifying cancer-related myths, and vice versa. One possible explanation is that individuals tend to focus on either scientific facts or popular beliefs, resulting in an imbalanced understanding of cancer risk. These findings point to a potential fragmentation in cancer-related health literacy and underscore the need for further investigation into the cognitive and informational factors contributing to this divide.

In the regression analyses, academic year, normal BMI, and oncology course attendance emerged as significant predictors of cancer risk factor awareness. Higher academic standing was positively associated with greater awareness of validated cancer risk factors, possibly reflecting the cumulative effect of academic exposure and health-related curricula. Similarly, students who had taken an oncology course demonstrated higher CAM scores, suggesting that structured education plays a crucial role in improving cancer literacy. Interestingly, normal BMI was linked to better awareness of evidence-based cancer risks, which may reflect the adoption of health-promoting behaviors informed by accurate knowledge. These findings align with previous literature that highlights the impact of education and personal health behaviors on cancer awareness levels [9,11,12]. However, the limited explanatory power of the models (R² = .142 for CAM and R² = .072 for CAM-MYCS) suggests that additional unmeasured factors—such as psychological, cultural, or informational influences—may also play a role. The regression model for CAM-MYCS revealed that lower grade level and higher BMI were significant predictors of increased awareness of mythical cancer risk factors. These findings suggest that younger students and those with higher body mass may be more susceptible to misinformation or less able to critically evaluate unvalidated health claims. The effect size, while modest (f² = 0.078), indicates a meaningful association worth addressing in future educational interventions targeting myth-related cancer beliefs.

## Limitations

This study has several limitations that should be considered when interpreting the findings. First, its cross-sectional design precludes the establishment of causal relationships between awareness of cancer risk factors and myths and participants’ sociodemographic characteristics or health and lifestyle behaviors. Additionally, the design does not allow for the evaluation of changes in awareness over time or the impact of educational interventions. Future research would benefit from longitudinal designs or pre-post assessments following awareness campaigns to evaluate intervention effectiveness.

Second, the study sample consisted exclusively of health sciences students from three universities. While the relatively large sample enhances internal validity, the restricted academic background may limit the generalizability of the findings. Including students from non-health disciplines in future research would allow for broader insights into cancer-related awareness across diverse educational contexts.

Third, the use of an online, self-reported survey may have introduced self-selection bias, as individuals with higher awareness or greater interest in cancer-related topics may have been more likely to participate. Moreover, the study did not account for external influences such as prior health education, family history, or exposure to health information via social media—all of which may shape awareness levels. Including these variables in future research could provide a more nuanced understanding of the factors influencing knowledge about cancer risk.

Fourth, although the sample included young adults of reproductive age, the study did not comprehensively assess awareness of prenatal cancer risk factors—such as maternal smoking, alcohol use, or micronutrient deficiencies—which may influence cancer susceptibility later in life. Future research should consider integrating these dimensions to inform early-life cancer prevention strategies.

Finally, while this study focused on awareness of validated and mythical cancer risk factors, it did not assess the psychological consequences of misinformation. Belief in unsubstantiated causes may contribute to unnecessary anxiety, distorted risk perception, or maladaptive health behaviors. Future studies should explore the emotional and behavioral impacts of cancer myths to inform more effective public health messaging. Moreover, it should be acknowledged that some factors currently perceived as mythical (e.g., certain genetically modified foods) may eventually be validated as genuine cancer risk factors as scientific knowledge evolves. Acknowledging this possibility reflects the importance of intellectual humility in health research.

## Conclusion

This study revealed that university students exhibited higher awareness of evidence-based cancer risk factors than of mythical causes. A negative correlation between awareness of validated and mythical risk factors was observed, yet awareness of myths remained low. Academic year, normal BMI, and oncology education were key factors associated with awareness levels.

These findings underscore the need for evidence-based educational strategies, especially for young adults. Integrating oncology-related topics into university curricula and supporting campus-level awareness programs may help correct misconceptions. Broader public campaigns are also essential to promote accurate knowledge and healthier behaviors.

## Supporting information

S1 Checklist STROBE (Strengthening the Reporting of Observational Studies in Epidemiolgy) checklist for cross-sectional studies. (DOCX)

S2 Survey Instrument Structured questionnaire used for data collection, including the Personel Information Form, CAM and CAM-MYCS items, and the Health and Lifestyle Behaviors Form. (DOCX)

S3 Dataset The dataset generated and analyzed during the current study, provided in.xlsx format. This file includes all raw data underlying the means, standart deviations, and other statistical values reported in the manuscript.
(XLSX)

S4 Grahp Data Excel file containing the numerical data used to construct all figures and graphs presented in the manuscript. (XLSX)
