# Supplementary material for: Understanding real and mythical cancer risk factors: Insights from a university-based study
Source: PLoS One. 2025 Nov 26;20(11):e0336102. doi: 10.1371/journal.pone.0336102 (PMC12654919; doi:10.1371/journal.pone.0336102)
Supplement: S1 Checklist — (DOCX) [file pone.0336102.s001.docx]

# STROBE Checklist

|  | Item No. | | Recommendation | Page  No. | | | Reported on Section | |
| --- | --- | --- | --- | --- | --- | --- | --- | --- |
| **Title and abstract** | 1 | | (*a*) Indicate the study’s design with a commonly used term in the title or the abstract | 2 | | | | Abstract |
|  |  |  | (*b*) Provide in the abstract an informative and balanced summary of what was done and what was found | 2 | | | | Abstract |
| Introduction | | | | | | | |  |
| Background/rationale | 2 | | Explain the scientific background and rationale for the investigation being reported | 3 | | | | Introduction |
| Objectives | 3 | | State specific objectives, including any prespecified hypotheses | 3-4 | | | | Introduction |
| Methods | | | | | | | |  |
| Study design | 4 | | Present key elements of study design early in the paper | 4 | | | | Materials and methods – Study Design and Setting |
| Setting | 5 | | Describe the setting, locations, and relevant dates, including periods of recruitment, exposure, follow-up, and data collection | 4-5 | | | | Materials and methods – Study Design and Setting |
| Participants | 6 | | (*a*) *Cohort study*—Give the eligibility criteria, and the sources and methods of selection of participants. Describe methods of follow-up  *Case-control study*—Give the eligibility criteria, and the sources and methods of case ascertainment and control selection. Give the rationale for the choice of cases and controls  *Cross-sectional study*—Give the eligibility criteria, and the sources and methods of selection of participants | 5  5 | | | Materials and methods - Inclusion and Exclusion Criteria | |
|  |  |  | (*b*) *Cohort study*—For matched studies, give matching criteria and number of exposed and unexposed  *Case-control study*—For matched studies, give matching criteria and the number of controls per case |  | | |  | |
| Variables | 7 | | Clearly define all outcomes, exposures, predictors, potential confounders, and effect modifiers. Give diagnostic criteria, if applicable | 5-7 | | | Materials and methods- Study variables and measurement tools | |
| Data sources/ measurement | 8* | | For each variable of interest, give sources of data and details of methods of assessment (measurement). Describe comparability of assessment methods if there is more than one group | 5-7 | | | Materials and methods- Study variables and measurement tools | |
| Bias | 9 | | Describe any efforts to address potential sources of bias | 5-6,22 | | | Materials and Methods – Sampling Procedure & Study Variables and Measurement Tools; also discussed in Limitations. | |
| Study size | 10 | | Explain how the study size was arrived at | 5 | | | Materials and Methods – Sample size and determination | |
| Quantitative variables | | 11 | Explain how quantitative variables were handled in the analyses. If applicable, describe which groupings were chosen and why | | 7,8-9 | Materials and Methods – Statistical Analysis (continuous variables analyzed directly; BMI, diet, and physical activity categorized per WHO guidelines) | | |
| Statistical methods | | 12 | (*a*) Describe all statistical methods, including those used to control for confounding | | 8-9 | **Materials and Methods – Statistical Analysis** | | |
|  |  |  | (*b*) Describe any methods used to examine subgroups and interactions | | 8-9, 13,14 | Statistical Analysis and Results | | |
|  |  |  | (*c*) Explain how missing data were addressed | | 5 | Materials and Methods (no missing data; complete case analysis applied) | | |
|  |  |  | (*d*) *Cohort study*—If applicable, explain how loss to follow-up was addressed  *Case-control study*—If applicable, explain how matching of cases and controls was addressed  *Cross-sectional study*—If applicable, describe analytical methods taking account of sampling strategy | | - | Not applicable | | |
|  |  |  | (*e*) Describe any sensitivity analyses | | - | Not applicable | | |
| Result | | | | | | | | |
| Participants | | 13* | (a) Report numbers of individuals at each stage of study—eg numbers potentially eligible, examined for eligibility, confirmed eligible, included in the study, completing follow-up, and analysed | | - | Only final sample size (n = 896) is reported; information on earlier recruitment stages was not collected. | | |
|  |  |  | (b) Give reasons for non-participation at each stage | | - | Not reported – Reasons for non-participation were not collected in the study. | | |
|  |  |  | (c) Consider use of a flow diagram | | - | Not included – A flow diagram was not deemed necessary given the simplicity of participant inclusion. | | |
| Descriptive data | | 14* | (a) Give characteristics of study participants (eg demographic, clinical, social) and information on exposures and potential confounders | | 9-10 | Results – Table 1 and Sociodemographic factors | | |
|  |  |  | (b) Indicate number of participants with missing data for each variable of interest | | 9-10 | All variables had complete data (n = 896); no missing data to report. | | |
|  |  |  | (c) *Cohort study*—Summarise follow-up time (eg, average and total amount) | | - | Not applicable | | |
| Outcome data | | 15* | *Cohort study*—Report numbers of outcome events or summary measures over time | |  |  | | |
|  |  |  | *Case-control study—*Report numbers in each exposure category, or summary measures of exposure | |  |  | | |
|  |  |  | *Cross-sectional study—*Report numbers of outcome events or summary measures | | *9-15* | Results-Tables, Figure 1 | | |
| Main results | | 16 | (*a*) Give unadjusted estimates and, if applicable, confounder-adjusted estimates and their precision (eg, 95% confidence interval). Make clear which confounders were adjusted for and why they were included | | 8-9; 14-15 | Methods and Results – Adjusted estimates derived from multiple linear regression including potential confounders (age, gender, academic year, BMI, lifestyle variables). | | |
|  |  |  | (*b*) Report category boundaries when continuous variables were categorized | | 5-7 | Materials and Methods – Study variables and measurement tools | | |
|  |  |  | (*c*) If relevant, consider translating estimates of relative risk into absolute risk for a meaningful time period | | - | Not applicable – This was a cross-sectional study; relative or absolute risk estimates were not calculated. | | |
| Other analyses | | 17 | Report other analyses done—eg analyses of subgroups and interactions, and sensitivity analyses | | 10-14, 8-9 | Results, Statistical Analysis | | |
| Discussion | | | | | | | | |
| Key results | | 18 | Summarise key results with reference to study objectives | | 16 | Discussion | | |
| Limitations | | 19 | Discuss limitations of the study, taking into account sources of potential bias or imprecision. Discuss both direction and magnitude of any potential bias | | 21-22 | Discussion, Limitations | | |
| Interpretation | | 20 | Give a cautious overall interpretation of results considering objectives, limitations, multiplicity of analyses, results from similar studies, and other relevant evidence | | 16-21 | Discussion | | |
| Generalisability | | 21 | Discuss the generalisability (external validity) of the study results | | 21,22 | Limitations | | |
| Other information | | |  | | | | | |
| Funding | | 22 | Give the source of funding and the role of the funders for the present study and, if applicable, for the original study on which the present article is based | | 23 | Financial disclosure statement **(No external funding; the funders had no role in study design, data collection, analysis, or interpretation)** | | |
