# Supplementary material for: Understanding real and mythical cancer risk factors: Insights from a university-based study
Source: PLoS One. 2025 Nov 26;20(11):e0336102. doi: 10.1371/journal.pone.0336102 (PMC12654919; doi:10.1371/journal.pone.0336102)
Supplement: S2 Survey Instrument — (DOCX) [file pone.0336102.s002.docx]

**Personal Information Form**

Dear Students,

It is important for the reliability of this research that you answer each question in the questionnaire sincerely and accurately. You are kindly requested to respond to all questions completely and truthfully.

Below, you will find the **Personal Information Form,** the **Cancer Awareness Questionnaire,** and the **Cancer Awareness Causes Scale**.

If you agree to participate in this study, please note that you are **not required to provide any personal identifying information** on the questionnaire. All data collected will be used **solely for research purposes**, and no institutional identifiers will be disclosed in any presentation or publication of the study results.

**I voluntarily agree to participate in this study.**

**I approve.**

**1. Your age………**

**2. Your gender?**

a. Female b. Male

**3.Your marital status?**

a. Married b. Single

**4. Which department are you studying in?**a. Nutrition and Dietetics
b. Child Development
c. Speech and Language Therapy
d. Midwifery
e. Nursing
f. Occupational Therapy
g. Physiotherapy and Rehabilitation
h. Audiology

**5.Your academic year?**

a. First year b. Second year c. Third year d. Fourth year

**6. How would you describe your income level?**

a. Low (0-25.000 Turkish Lira (₺)) b. Middle (25.001 ₺-60.000 ₺

c. High (60.001 ₺ and above)

**7. Have you taken any courses related to oncology?**

a. Yes b. No

**8.** **Do you have a relative who has been diagnosed with cancer?**

a. Yes b. No

**Cancer Awareness Measure (CAM)**

| **Questions** | **1** | **2** | **3** | **4** | **5** |
| --- | --- | --- | --- | --- | --- |
|  | **Strongly Agree** | **Agree** | **Neutral** | **Disagree** | **Strongly Disagree** |
| 1. Active smoking is a risk factor for cancer. |  |  |  |  |  |
| 2. Alcohol consumption is a risk factor for cancer. |  |  |  |  |  |
| 3. Passive smoking is a risk factor for cancer. |  |  |  |  |  |
| 4. Sunburns are a risk factor for cancer. |  |  |  |  |  |
| 5. Human Papilloma Virus (HPV) is a risk factor for cancer. |  |  |  |  |  |
| 6. Processed red meat products are a risk factor for cancer. |  |  |  |  |  |
| 7. Obesity is a risk factor for cancer. |  |  |  |  |  |
| 8. Having a relative diagnosed with cancer is a risk factor for cancer. |  |  |  |  |  |
| 9. Inadequate physical activity (exercise) is a risk factor for cancer. |  |  |  |  |  |
| 10. Aging is a risk factor for cancer. |  |  |  |  |  |
| 11. Inadequate fruit and vegetable consumption is a risk factor for cancer. |  |  |  |  |  |

**Cancer Awareness Measure – Mythical (Unproven) Causes Scale (CAM-MYCS)**

| **Questions** | **1** | **2** | **3** | **4** | **5** |
| --- | --- | --- | --- | --- | --- |
|  | **Strongly Agree** | **Agree** | **Neutral** | **Disagree** | **Strongly Disagree** |
| **1. Exposure to electromagnetic frequencies (such as Wi-Fi, television, and radio) is a risk factor for cancer.** |  |  |  |  |  |
| **2. Eating foods that contain additives is a risk factor for cancer.** |  |  |  |  |  |
| **3. Living near power lines is a risk factor for cancer.** |  |  |  |  |  |
| **4. Being under stress is a risk factor for cancer.** |  |  |  |  |  |
| **5. Consuming foods with artificial sweeteners is a risk factor for cancer.** |  |  |  |  |  |
| **6. Using cleaning products is a risk factor for cancer.** |  |  |  |  |  |
| **7. Using a mobile phone is a risk factor for cancer.** |  |  |  |  |  |
| **8. Eating genetically modified foods (GMF) is a risk factor for cancer.** |  |  |  |  |  |
| **9. Using aerosol spray cans is a risk factor for cancer.** |  |  |  |  |  |
| **10. Physical traumas such as being hit or crushed are risk factors for cancer.** |  |  |  |  |  |
| **11. Using a microwave oven is a risk factor for cancer.** |  |  |  |  |  |
| **12. Drinking from plastic bottles is a risk factor for cancer.** |  |  |  |  |  |

**Health and Lifestyle Behaviors Form**

**1. Your height……….**

**2. Your weight……….**

**3. Do you smoke?**

a. Yes b. No

**4. On how many days during the past week did you engage in physical activity that increased your breathing rate for at least 30 minutes in total?**

a. I do not exercise b.1-2 days c. 3-4 days d.5 or more days

**5. During the past month, how many portions (1 portion = 80 g; e.g., one small apple ≈ 90 g) of fruits and vegetables did you consume per day?**

a. 1-2 portions b. 3-4 portions c. 5 or more portions

**6. Do you consume alcohol?**

a. Yes b. No

**7.** **If you consume alcohol, on how many days per week do you usually drink?**

a. 1 day b. 2 day c. 3 day d.4 day e. 5 day

f. 6 day e. 7 day

**8. How many units of alcohol do you typically consume per day?
(One unit = one bottle or can of beer, one glass of wine, or 45 ml of strong spirits such as vodka or whiskey.)**

a. 1-2 units b. 3 units c. 4 units d. 5 units e. 6 units f. 7 units
